# Neonate Born to a Mother with a Diagnosis of Suspected Intra-Amniotic Infection versus COVID-19 or Both

**DOI:** 10.1155/2020/8886800

**Published:** 2020-07-18

**Authors:** Rishi Lumba, Juan Remon, Moi Louie, Michelle Quan, Sourabh Verma, Mona Rigaud, Bgee Kunjumon

**Affiliations:** Department of Pediatrics, New York University Grossman School of Medicine, New York, NY, USA

## Abstract

A diagnosis of intra-amniotic infection is typically made based on clinical criteria, including maternal intrapartum fever and one or more of the following: maternal leukocytosis, purulent cervical drainage, or fetal tachycardia. The diagnosis can also be made in patients with an isolated fever of 39°C, or greater, without any other clinical risk factors present. Coronavirus disease 2019 (COVID-19), caused by the virus SARS-CoV-2, has been noted to have varying signs and symptoms over the course of the disease including fever, cough, fatigue, anorexia, shortness of breath, sputum production, and myalgia. In this report, we detail a case of a newborn born to a mother with a clinical diagnosis of intra-amniotic infection with maternal fever and fetal tachycardia, who was then found to be SARS-CoV-2 positive on testing. Due to the varying presentation of COVID-19, this case illustrates the low threshold needed to test mothers for SARS-CoV-2 in order to prevent horizontal transmission to neonates and to healthcare providers.

## 1. Introduction

Intra-amniotic infection, also known as chorioamnionitis or more recently as intra-amniotic infection and inflammation (Triple I) [1], is a relatively common condition in pregnant mothers. Approximately 2% to 5% of term deliveries are complicated with a clinically apparent intra-amniotic infection [2, 3] with most cases of intra-amniotic infection diagnosed clinically by the obstetrics team. The current recommendations made by the American College of Obstetricians and Gynecologists (ACOG) are that the diagnosis of suspected intra-amniotic infection be made on clinical criteria, which include maternal intrapartum fever and one or more of the following: maternal leukocytosis, purulent cervical drainage, or fetal tachycardia. Additionally, ACOG recommends a diagnosis of suspected intra-amniotic infection be made in patients with an isolated maternal fever of 39°C, or greater, without any other clinical risk factors present [4].

In December 2019, China reported a cluster of pneumonia cases caused by a novel pathogenic coronavirus, subsequently named Severe Acute Respiratory Syndrome Coronavirus 2 (SARS-CoV-2) and, on March 11, 2020, the World Health Organization declared COVID-19 a global pandemic. The infection has spread worldwide to more than 200 countries, with over three million individuals testing positive and resulting in over two hundred thousand deaths. Signs and symptoms of COVID-19 vary over the course of the disease and include fever, cough, fatigue, anorexia, shortness of breath, sputum production, and myalgia [5–7]. Studies have also shown that SARS-CoV-2 infection can occur in asymptomatic and presymptomatic patients [8–10]. Due to the concern that there are many asymptomatic cases and due to the overlapping signs and symptoms of COVID-19 with maternal Triple I, some experts have recommended testing every mother coming to the hospital for a delivery [11].

We report a case of a newborn born to a mother who was clinically diagnosed with suspected Triple I, but then subsequently confirmed to have COVID-19.

## 2. Case Presentation

A full-term infant weighing 3.11 kg was born at 39 weeks and 4 days gestation age via normal spontaneous vaginal delivery, to a healthy 20-year-old G2P1011 mother. Maternal fever of 38.8°C (101.9°F) was noted 3 hours prior to delivery, with associated fetal tachycardia. However, no other symptoms were noted and maternal history and prenatal labs, including Group B streptococcus, were unremarkable. Spontaneous rupture of membranes with clear amniotic fluid was noted 8 hours prior to delivery. A neonatal team attended the delivery, and routine newborn care was given with Apgar score 9 at both 1 and 5 minutes, respectively.

Our institutional policy at the time was to have a low threshold for maternal SARS-CoV-2 testing in pregnant mothers, including any who developed an isolated fever. Although a clinical diagnosis of Triple I was made by the obstetrics team, given maternal fever, testing for SARS-CoV-2 was included as well. Appropriate personal protective equipment (PPE) was donned and doffed before and after delivery. Additionally, the newborn was admitted to the NICU and was placed in appropriate isolation as a person under investigation (PUI) for COVID-19 and for presumed infection due to maternal Triple I. Even though the mother had a clinical diagnosis of Triple I and no other symptoms except for fever, she later tested positive for SARS-CoV-2.

In the NICU, a partial sepsis workup was initiated, as per our guidelines for the management of asymptomatic neonates born to mothers with suspected chorioamnionitis. The neonate was treated with broad-spectrum antibiotics, ampicillin and gentamicin, after drawing a blood culture. A complete blood count test with manual differential at six hours of life was sent as per protocol. A white cell count of 16,000 per mm^3^ and platelets of 168,000 per mm^3^ were found, and on manual differential, there were 72 neutrophils and an immature to total neutrophil ratio of 0.04. The results of the physical exam and vitals were normal throughout the admission. Antibiotics were discontinued after the blood culture was negative for 48 hours. The neonate was tested for SARS-CoV-2 by nasopharyngeal swab using a real-time reverse transcriptase polymerase chain reaction at birth, and before discharge, as per local institutional policy for testing SARS-CoV-2 in neonates born to mothers with COVID-19. The results were negative at both occasions. Due to the maternal SARS-CoV-2 status, the patient was discharged home to his father with anticipatory guidance for routine care and infection prevention.

## 3. Discussion

The above case illustrates the difficulty in a diagnosis of Triple I during the COVID-19 pandemic. In this case, the only symptom the mother had was a fever without any other signs and symptoms of COVID-19 including cough, fatigue, anorexia, shortness of breath, sputum production, or myalgia. Due to our low testing threshold for SARS-CoV-2, the mother was subsequently diagnosed as having COVID-19. Our case should draw attention to the possibility of a potential postnatal exposure to SARS-CoV-2 in febrile mothers suspected to have Triple I as an alternative diagnosis or as a coinfection, and it is important that both should be considered in the clinical decision-making process. Maternal testing, when available, should be offered to screen women at increased risk during the pandemic. The chances of vertical transmission of SARS-CoV-2 from mother to infant based upon current limited published data seem low [12, 13]; however, due to the close contact between mothers and infants after delivery, the possibility of horizontal infection should be mitigated. Appropriate identification of mothers infected with SARS-CoV-2 is also important in prevention of transmission spread of SARS-CoV-2 to healthcare professionals and to other neonates if the mother were to visit the NICU.

Though difficult, until a negative confirmatory test result is obtained, separation of the mother and newborn after birth will minimize the risk of postnatal infant infection. Isolation from the mother, when possible, and other neonates would also be recommended to prevent spread to other newborns. Given the potential asymptomatic spread of SARS-CoV-2, universal screening of mothers predelivery should also be considered, depending on available resources, to prevent unnecessary separation of infants and to prevent neonatal infection and the spread of COVID-19 to staff and families. Our institution has therefore now changed its policy to universally test all pregnant mothers on admission.

## Data Availability

The clinical and laboratory data used to support the findings of this study are included within the article.
